# Inequitable impact of infection: social gradients in severe COVID-19 outcomes among all confirmed SARS-CoV-2 cases during the first pandemic wave in Sweden

**DOI:** 10.1136/jech-2021-216778

**Published:** 2021-09-14

**Authors:** Per E. Gustafsson, Miguel San Sebastian, Osvaldo Fonseca-Rodriguez, Anne-Marie Fors Connolly

**Affiliations:** 1 Department of Epidemiology and Global Health, Umeå University, Umeå, Sweden; 2 Department of Clinical Microbiology, Umeå University, Umeå, Sweden

**Keywords:** cohort studies, health inequalities, COVID-19, directory, social epidemiology

## Abstract

**Background:**

The backdrop of the ubiquitous social inequalities has increasingly come into foreground in research on the COVID-19 pandemic, but the lack of high-quality population-based studies limits our understanding of the inequitable outcomes of the disease. The present study seeks to estimate social gradients in COVID-19 hospitalisations, intensive care admissions and death by education, income and country of birth, while taking into account disparities in comorbidities.

**Methods:**

We used a register-based retrospective open cohort design enrolling all 74 659 confirmed SARS-CoV-2-positive cases aged >25 years in Sweden during the first wave of the pandemic (until 14 September 2020). Information was retrieved from multiple registers and linked by the unique Swedish personal identity number concerning COVID-19 case identification; COVID-19 hospitalisations, intensive care admissions and death; comorbidities as measured by the Charlson Comorbidity Index; and sociodemographic information. Social gradients were estimated by the Relative Index of Inequality (RII) using Cox regression.

**Results:**

Adjusted analyses showed significant social gradients in COVID-19 hospitalisation, intensive care admission, across education, income and country of birth, which were unaffected by adjustment for comorbidities. Education and country of birth gradients were stronger for hospitalisation and intensive care admissions but small to non-existent for death. In contrast, income gradients were consistent across all three COVID-19 outcomes.

**Conclusion:**

Social gradients in severe COVID-19 outcomes are widespread in Sweden, but appear to be unrelated to pre-existing health disparities. Inequitable outcomes of SARS-CoV-2 infection may therefore be at least partially avoidable and could rely on equitable management of confirmed COVID-19 cases.

## Introduction

After the immediate shock when the COVID-19 pandemic emerged in 2020, concerns were raised about the impact of the already entrenched social inequalities in health on the course of the pandemic.^1 2^ Initial empirical COVID-19 research largely overlooked this issue,^3^ which risks leaving a permanent lack of the crucial data needed to detail the socioeconomic patterning of COVID-19 in the early stage of the pandemic.^3 4^

Sweden, with its infrastructure of individually linked register data, is in a strong position to shed light on social inequalities in COVID-19. The Swedish Constitution precluded enforcement of general lockdown measures implemented in many other countries during the first pandemic wave. Sweden instead opted for an unconventional strategy to mitigate rather than stop the pandemic, to avoid overburdening the healthcare system.^5^ The response chiefly relied on voluntary measures, including recommendations of social distancing and working from home, but with no stay-at-home orders or recommendations to wear face masks in public. Schools for age 17 years and older largely adapted to distance education, while kindergarten and schools up to age 16 years remained open. Limitations were also imposed on public gatherings and restaurants, but with public space, stores and services kept open. This comparatively non-invasive pandemic strategy has been the topic of an intense international debate.^6–8^ Sweden’s general public health policy also explicitly stresses equity in health and healthcare^9^ and prioritised COVID-19 vaccination for individuals in socially vulnerable situations.^10^ Sweden is therefore an informative case for countries aiming for equitable pandemic strategies under less restrictive regimes.

Preliminary research, chiefly based on samples from the USA and UK, overall points to an unequal social distribution of both risk for COVID-19 infection,^11^ hospital or intensive care admissions, or death.^12–14^ Most attention has so far been paid to ethnic/racial inequalities^12^ but with studies also suggesting a socioeconomic gradient in COVID-19 outcomes.^11 13–16^ Population-based studies on individual-level data remains rare. Two register-based total population studies of Sweden^17^ and the capital region of Stockholm^18^ have shown higher COVID-19 mortality among groups with lower income, shorter education and particularly those born in low-income (LIC) and middle-income countries (MIC). These findings support earlier Swedish ecological research reporting excess COVID-19 mortality in disadvantaged neighbourhoods.^16^ These studies were however unable to further detail the potential underpinnings of the mortality inequalities: differential exposure to the virus, differential immunity and/or differential consequences once infected—including severe COVID-19 disease and survival.^19^ The present study seeks to explore the lattermost possibility.

A widespread hypothesis is that social inequalities in severe COVID-19 outcomes are rooted in pre-existing health risks,^2 17 19 20^ as chronic diseases identified as risk factors for severe COVID-19 outcomes^10 21^ tend to follow the familiar social gradient in health.^22^ Preliminary studies from the USA^23^ and UK^14^ have indeed suggested that comorbidities partially explain ethnic inequalities in COVID-19 hospitalisations. A second not mutually exclusive hypothesis is that disadvantaged population groups fare worse because of inequitable healthcare.^12 20^ This has, for example, been suggested by findings of inequalities in COVID-19 hospitalisations in the USA, which remain after consideration of pre-existing comorbidities.^24 25^ Evidence on these explanations is crucial for the design of equitable policy: while support for the former hypothesis would leave little room for immediate action, support for the second would turn the spotlight to health system’s management of patients with COVID-19.

The aim of the present study is to estimate the magnitude of educational, income and country of birth gradients in COVID-19 hospitalisations, intensive care admissions and death in the total Swedish population of SARS-CoV-2-positive adults of the first wave of the pandemic, taking into account pre-existing comorbidities.

## Methods

### Population and design

The study used a register-based retrospective open cohort design, enrolling all Swedish residents aged 26 years or older who tested positive for SARS-CoV-2 between 4 February and 14 September 2020, comprising the entire first wave of the pandemic in Sweden (approximately between March and August 2020^26^). Individuals aged ≤25 were excluded as at this age a large fraction is still studying or has not yet entered the labour market.

Case-positive individuals were identified through SmiNet, the register of notifiable diseases at the Public Health Agency of Sweden. COVID-19 is a notifiable disease in Sweden, and PCR-positive or antigen positive SARS-CoV-2 cases are reported by the treating clinicians to SmiNet. The date of COVID-19 was generated based on the earliest possible date available from the following five options, assessed in order from option (1) through (5): date of (1) disease onset, as indicated by the first symptoms experienced by the patient; (2) contact with inpatient or outpatient clinics due to COVID-19; (3) blood sample drawn for laboratory confirmation; (4) laboratory-confirmed diagnosis; or (5) case reported to the Public Health Agency. Individuals were followed from the date of COVID-19 diagnosis to date of hospitalisation (data until 22 September), intensive care (data until 12 August) and death (data until 5 October). Cases with outcomes preceding test positivity date were excluded. All individual data from the different data sources were linked through the unique Swedish personal identity number.

The cohort comprised N=74 659 individuals at risk with total follow-up time amounting to 6 312 229 days (mean follow-up time 85.2 days) for hospitalisation; 5 514 897 days (mean 78.6 days) for intensive care admissions; and 8 877 016 days (mean 118.9 days) for death. Certain variables had incomplete data, including comorbidities (n=1451), housing (n=2553, coded as separate category), family structure (n=297), marital status (n=195), education (n=1624), income (n=537) and place of birth (n=24). This resulted in a total sample size of N=72 728, with analytical samples of N=72 145 for hospitalisation and death, and N=67 816 for intensive care admissions.

### Measures

#### COVID-19 outcomes

Time to event (days) from COVID-19 disease to endpoint was calculated for three indicators of severe COVID-19 disease: *hospitalisation*, *intensive care* and *death*. Outcomes were identified through National Inpatient Register and Cause of Death Register of the National Board of Health and Welfare, and through The Swedish Intensive Care Register, which is the National Quality Registry for Intensive Care in Sweden. COVID-19 main or contributing diagnosis, or underlying cause of death, was identified by ICD-10 code U07.

#### Inequality indicators

Sociodemographic information was retrieved from population registers of Statistics Sweden. The main socioeconomic variables comprised *education*, classified into primary, secondary and tertiary education; *income*, based on annual disposable income and divided into quintiles (Q1—poorest, Q5—richest); and *country of birth*, classified into Sweden, high-income country (HIC), middle-income country (MIC) and low-income country (LIC) based on Gross National Income per capita.^27^ For the purpose of the main analyses, all variables were treated as ordinal.

#### Covariates

Risk factors for severe COVID-19 outcomes included *sex* (man/woman) and *age* (years, in brackets) from population registers of Statistics Sweden. Comorbidities were measured by the *Charlson Comorbidity Index* (CCI), which has been validated for severe COVID-19 outcomes.^21^ We used a recent adaptation of the CCI developed for register-based research in Sweden^28^ based on data from the National Inpatient and Outpatient Registers and the Cancer Register of Sweden. The CCI comprises a weighted summary score calculated from a range of chronic diseases (see Ludvigsson *et al* 28 for details). In order to avoid including acute complications of COVID-19, only comorbidities identified ≥60 days prior to the test-positivity date were considered. The continuous comorbidity score (range 0–21) was used in the regression analyses, while in table 1 it is reported by categories.

**Table 1:** Descriptive statistics in the total sample and stratified by sex

	n (%)	Men (%)	Women (%)
Sample size	72 728 (100.00)	30 797 (42.35)	41 931 (57.65)
Age groups			
26–45	26 537 (36.49)	10 661 (34.62)	15 876 (37.86)
46–55	15 626 (21.49)	6286 (20.41)	9340 (22.27)
56–65	12 166 (16.73)	5345 (17.36)	6821 (16.27)
66–75	5713 (7.86)	3283 (10.66)	2430 (5.80)
76–85	6091 (8.38)	3007 (9.76)	3084 (7.35)
86–95	5674 (7.80)	1983 (6.44)	3691 (8.80)
=>96	921 (1.27)	232 (0.75)	689 (1.64)
Comorbidity index			
0	41 329 (57.80)	17 148 (57.32)	24 181 (58.15)
1–2	15 059 (21.06)	6217 (20.78)	8848 (21.26)
3–4	5514 (7.71)	2593 (8.67)	2921 (7.02)
=>5	9600 (13.43)	3959 (13.23)	5641 (13.56)
Housing			
House	31 014 (42.64)	12 969 (42.11)	18 045 (43.03)
Flat/semidetached	34 932 (48.03)	15 270 (49.58)	19 662 (46.89)
Other	997 (1.37)	442 (1.44)	555 (1.32)
Special accommodation	3261 (4.98)	1226 (3.98)	2395 (5.71)
Missing	2164 (2.98)	890 (2.89)	1274 (3.04)
Family structure			
Together w/out children	16 641 (22.88)	7740 (25.13)	8901 (21.23)
Together with children	23 947 (32.93)	10 148 (32.95)	13 799 (32.91)
Alone without children	18 147 (24.95)	7544 (24.504)	10 603 (25.29)
Alone with children	4759 (6.54)	1063 (3.45)	3696 (8.81)
Other without children	3922 (5.39)	1990 (6.46)	1932 (4.61)
Other with children	5312 (7.30)	2312 (7.51)	3000 (7.15)
Marital status			
Partner	32 876 (45.20)	14 974 (48.62)	17 902 (42.69)
Single	22 434 (30.85)	10 087 (32.75)	12 347 (29.45)
Widowed	6384 (8.78)	1551 (5.04)	4833 (11.53)
Divorced	11 034 (15.17)	4185 (13.59)	6849 (16.33)
Region of residence			
Other	36 555 (50.26)	14 718 (47.79)	21 837 (52.08)
Västra götaland	15 784 (21.70)	6955 (22.58)	8829 (21.06)
Stockholm	20 389 (28.03)	9124 (29.63)	11 265 (26.87)
Education			
Tertiary	29 247 (40.21)	11 375 (36.94)	17 872 (42.62)
Secondary	30 677 (42.18)	13 422 (43.58)	17 255 (41.15)
Primary	12 804 (17.61)	6000 (19.48)	6804 (16.23)
Income			
Quintile 1 (richest)	14 838 (20.40)	7007 (22.75)	7831 (18.68)
Quintile 2	14 827 (20.39)	6477 (21.03)	8350 (19.91)
Quintile 3	14 804 (20.36)	6064 (19.69)	8740 (20.84)
Quintile 4	14 602 (20.08)	5944 (19.30)	8658 (20.65)
Quintile 5 (poorest)	13 657 (18.78)	5305 (17.23)	8532 (19.92)
Place of birth			
Sweden	52 201 (71.78)	21 578 (70.07)	30 623 (73.03)
High-income country	5427 (7.46)	2138 (6.94)	3289 (7.84)
Middle-income country	11 040 (15.18)	5062 (16.44)	5978 (14.26)
Low-income country	4060 (5.58)	2019 (6.56)	2041 (4.87)
Outcomes			
Hospitalisation	16 807 (23.11)	9634 (31.28)	7173 (17.11)
Intensive care	2735 (3.76)	1926 (6.25)	809 (1.93)
Death	5863 (8.06)	3196 (10.38)	2667 (6.36)

Demographic covariates that can covary with COVID-19 outcomes^17 18 29^ were retrieved from Statistics Sweden and included *marital status* (married/cohabitation; unmarried/single; widowed; divorced), *type of residence* (house; apartment/semidetached; special accommodation (eg, nursing home); other), *household structure* (living alone with or without children; with spouse/partner with or without children with or without children; and missing data) and *region of residence* categorised by the largest urban regions with a concentration of COVID-19 cases (Stockholm; Västra Götaland; all other regions).

### Statistical analysis

Preliminary analyses comprised Cox regression for estimation of HRs with each COVID-19 outcome regressed on each exposure in crude/bivariate and fully adjusted models. In these analyses, the social inequality indicators were treated as categorical with the most favourable position as the reference group.

The Relative Index of Inequality (RII) was estimated as a relative measure of social gradients in health,^30 31^ which considers the population distribution of health across the social categories. It was estimated by fitting a regression slope to the midpoints of the cumulative distribution of time-to-event across the ranked categories of each inequality indicator (the *ridit* score). The RII is consistently expressed as a HR estimated from the regression slope at ridit score 1 vs 0; that is, a social gradient expressed as the relative hazard rate of the theoretically most disadvantaged group compared with the theoretically most advantaged group.

The RII (with 95% CI) was estimated for each outcome by each inequality indicator in five sequential models with exposure variables entered cumulatively: no adjustments (Model 0), adding the other two inequality indicators model (Model 1), adding age and sex (Model 2), adding the CCI (Model 3) and adding demographic covariates in a fully adjusted model (Model 4).

Supplementary analyses (see online supplemental supplement 1) were done restricting the analyses to deaths until 7 May (online supplemental table 1S) to conform to previous Swedish studies capturing approximately the first half of the first pandemic wave^17 18^ (online supplemental table 1S) and to ascertain the social distribution of comorbidities (online supplemental table 2S).

10.1136/jech-2021-216778.supp1 Supplementary data

## Results

Descriptive characteristics of the study population are shown in table 1. Of 72 728 individuals, 16 807 (23.1%) were hospitalised, 2735 (3.8%) were admitted to intensive care and 5863 (8.1%) died due to COVID-19.

Table 2 shows the HRs for COVID-19 hospitalisation, intensive care admission and death, respectively, by all exposure variables. Rates were higher among men, older people and those with greater comorbidity score, although the higher rates of hospitalisation and intensive care admissions levelled off among the oldest old. Of the demographic covariates, most notably was lower rates of hospitalisation and intensive care admissions, but higher death rates, among those living in special accommodations. Those with shortest education, lowest income and from a LIC overall displayed the highest hospitalisation and intensive care rates, and with overall patterns conforming to a social gradient in health. A clear social patterning of deaths was however only discernable for income. In supplementary analyses restricting the analysis to deaths until 7 May (online supplemental supplement, table 1S), higher death rates were also found among individuals from LIC. Supplementary analyses also confirmed that low-educated and poorer groups had a higher comorbidity score, but with no association found for country of birth (online supplemental supplement table 2S).

**Table 2:** Crude and fully adjusted incidence HRs with 95% CIs for COVID-19 hospitalisation, intensive care admissions and deaths

	Hospitalisations		Intensive care admissions		Deaths	
	Crude HR	Adjusted HR	Crude HR	Adjusted HR	Crude HR	Adjusted HR
Sex						
Men	1	1	1	1	1	1
Women	0.50 (0.49 to 0.52)	0.57 (0.55 to 0.58)	0.29 (0.27 to 0.32)	0.36 (0.33 to 0.39)	0.60 (0.57 to 0.63)	0.51 (0.48 to 0.54)
Age						
56–65	1	1	1	1	1	1
26–45	0.30 (0.29 to 0.32)	0.32 (0.30 to 0.34)	0.19 (0.16 to 0.21)	0.20 (0.18 to 0.23)	0.06 (0.05 to 0.09)	0.07 (0.05 to 0.10)
46–65	0.58 (0.55 to 0.61)	0.64 (0.61 to 0.68)	0.51 (0.46 to 0.57)	0.59 (0.52 to 0.66)	0.27 (0.21to 0.34)	0.30 (0.24 to 0.38)
66–75	2.22 (2.11 to 2.33)	1.67 (1.58 to 1.76)	1.93 (1.74 to 2.14)	1.40 (1.25 to 1.56)	6.40 (5.59 to 7.33)	4.28 (3.71 to 4.92)
76–85	2.36 (2.25 to 2.48)	1.76 (1.67 to 1.88)	0.79 (0.70 to 0.90)	0.60 (0.51 to 0.69)	15.71 (13.86 to 17.81)	8.84 (7.70 to 10.16)
86–95	1.54 (1.45 to 1.62)	1.38 (1.29 to 1.48)	0.14 (0.11 to 0.18)	0.13 (0.09 to 0.17)	22.17 (19.59 to 25.10)	12.39 (10.74 to 14.30)
=>96	0.95 (0.83 to 1.09)	1.00 (0.86 to 1.16)	0.09 (0.04 to 0.20)	0.10 (0.04 to 0.23)	26.72 (22.98 to 31.06)	14.47 (12.18 to 17.18)
Comorbidity index						
	1.06 (1.06 to 1.07)	1.03 (1.02 to 1.03)	1.04 (1.04 to 1.05)	1.03 (1.02 to 1.04)	1.13 (1.12 to 1.13)	1.03 (1.02 to 1.03)
Housing						
House	1	1	1	1	1	
Flat/semidetached	1.57 (1.52 to 1.62)	1.16 (1.11 to 1.20)	1.20 (1.10 to 1.30)	1.04 (0.95 to 1.14)	1.63 (1.53 to 1.74)	1.12 (1.04 to 1.20)
Other	1.52 (1.34 to 1.72)	1.05 (0.92 to 1.19)	1.25 (0.93 to 1.69)	1.10 (0.81 to 1.49)	2.24 (1.82 to 2.74)	1.16 (0.94 to 1.42)
Special accommodation	0.95 (0.87 to 1.03)	0.29 (0.27 to 0.32)	0.34 (0.26 to 0.46)	0.32 (0.24 to 0.43)	9.85 (9.14 to 10.61)	1.41 (1.30 to 1.54)
Missing	0.98 (0.89 to 1.09)	0.49 (0.44 to 0.54)	0.94 (0.75 to 1.19)	0.92 (0.72 to 1.17)	4.77 (4.28 to 5.31)	1.52 (1.36 to 1.70)
Family structure						
Together w/out children	1	1	1	1	1	1
Together with children	0.45 (0.43 to 0.47)	0.86 (0.82 to 0.91)	0.55 (0.49 to 0.61)	0.84 (0.75 to 0.95)	0.09 (0.08 to 0.10)	0.79 (0.67 to 0.28)
Alone without children	1.00 (0.96 to 1.04)	1.10 (1.05 to 1.17)	0.72 (0.65 to 0.80)	0.99 (0.87 to 1.13)	1.97 (1.85 to 2.09)	1.19 (1.08 to 1.31)
Alone with children	0.52 (0.48 to 0.56)	0.91 (0.84 to 0.99)	0.51 (0.42 to 0.62)	0.93 (0.75 to 1.15)	0.24 (0.20 to 0.29)	1.22 (1.00 to 1.48)
Other without children	0.79 (0.74 to 0.85)	0.87 (0.81 to 0.94)	0.91 (0.77 to 1.07)	1.07 (0.90 to 1.27)	0.91 (0.81 to 1.02)	1.16 (1.02 to 1.31)
Other with children	0.80 (0.75 to 0.85)	0.98 (0.92 to 1.05)	0.93 (0.80 to 1.07)	1.07 (0.91 to 1.25)	0.34 (0.29 to 0.40)	1.19 (1.01 to 1.40)
Civil status						
Partner	1	1	1	1	1	1
Single	0.51 (0.49 to 0.54)	0.82 (0.78 to 0.87)	0.60 (0.55 to 0.67)	0.95 (0.84 to 1.07)	0.48 (0.44 to 0.53)	0.88 (0.79 to 0.99)
Widowed	1.58 (1.51 to 1.65)	0.94 (0.88 to 1.00)	0.53 (0.46 to 0.63)	1.00 (0.83 to 1.22)	6.47 (6.09 to 6.88)	0.95 (0.86 to 1.05)
Divorced	1.22 (1.17 to 1.28)	0.93 (0.88 to 0.98)	1.02 (0.92 to 1.13)	0.89 (0.79 to 1.00)	1.64 (1.52 to 1.76)	0.85 (0.77 to 0.94)
Region of residence						
Other	1	1	1	1	1	1
Västra Götaland	0.75 (0.72 to 0.79)	0.79 (0.75 to 0.83)	0.98 (0.89 to 1.09)	0.99 (0.89 to 1.09)	0.81 (0.75 to 0.87)	0.93 (0.86 to 1.00)
Stockholm	1.80 (1.74 to 1.86)	1.58 (1.53 to 1.64)	1.10 (1.01 to 1.20)	0.93 (0.85 to 1.01)	1.63 (1.54 to 1.72)	1.21 (1.14 to 1.28)
Education						
Tertiary	1	1	1	1	1	1
Secondary (10–12 years)	1.48 (1.42 to 1.54)	1.18 (1.13 to 1.22)	1.56 (1.42 to 1.71)	1.25 (1.14 to 1.37)	1.98 (1.85 to 2.13)	1.12 (1.04 to 1.20)
Primary (<10 years)	2.92 (2.80 to 3.04)	1.40 (1.33 to 1.46)	2.14 (1.93 to 2.37)	1.35 (1.21 to 1.51)	5.41 (5.04 to 5.81)	1.11 (1.02 to 1.19)
Income						
Quintile 1 (richest)	1	1	1	1	1	1
Quintile 2	0.97 (0.92 to 1.02)	1.12 (1.06 to 1.18)	0.93 (0.83 to 1.06)	1.07 (0.95 to 1.22)	0.87 (0.76 to 0.99)	1.12 (0.98 to 1.27)
Quintile 3	1.06 (1.01 to 1.12)	1.20 (1.13 to 1.27)	0.91 (0.80 to 1.03)	1.15 (1.01 to 1.31)	1.58 (1.41 to 1.76)	1.35 (1.20 to 1.51)
Quintile 4	1.69 (1.60 to 1.77)	1.40 (1.33 to 1.48)	1.00 (0.88 to 1.12)	1.23 (1.07 to 1.41)	4.49 (4.08 to 4.94)	1.48 (1.33 to 1.64)
Quintile 5 (poorest)	2.05 (1.95 to 2.15)	1.58 (1.49 to 1.68)	1.45 (1.30 to 1.63)	1.51 (1.32 to 1.73)	4.49 (4.07 to 4.94)	1.62 (1.45 to 1.81)
Place of birth						
Sweden	1	1	1	1	1	1
High-income countries	1.63 (1.56 to 1.73)	1.20 (1.14 to 1.27)	1.34 (1.17 to 1.54)	1.14 (0.99 to 1.31)	1.36 (1.25 to 1.48)	1.04 (0.96 to 1.13)
Middle-income countries	1.54 (1.48 to 1.60)	1.59 (1.52 to 1.66)	1.70 (1.55 to 1.87)	1.43 (1.28 to 1.60)	0.33 (0.29 to 0.37)	0.85 (0.75 to 0.96)
Low-income countries	1.67 (1.57 to 1.77)	1.66 (1.55 to 1.78)	2.08 (1.83 to 2.37)	1.71 (1.47 to 1.98)	0.35 (0.29 to 0.41)	1.17 (0.97 to 1.40)

These descriptive patterns of social gradients were further analysed by estimation of RII, as displayed in table 3. The crude gradients (Model 0) showed diverse patterns which changed majorly after mutual adjustment for the other two socioeconomic indicators (Model 1) and for age and sex (Model 2). In Model 2, the majority of estimated gradients ranged between RII=1.5–2, that is, social gradients corresponding to 1.5–2 times higher rate of COVID-19 outcomes among those in the most disadvantaged compared with the most advantaged position. The gradients were stronger for hospitalisation by country of birth (RII=3.13) and income (RII=2.18), while the gradients in death were weaker for education and country of birth (RII=1.08–1.09).

**Table 3:** Social gradients of COVID-19-related hospitalisation, intensive care and death by education, income and country of birth; estimates are Relative Index of Inequality (RII) with 95% CIs based on Cox regression models*

	Model 0	Model 1	Model 2	Model 3	Model 4
Hospitalisations					
Education	4.29 (4.04 to 4.56)	3.33 (3.12 to 3.54)	1.47 (1.38 to 1.56)	1.45 (1.36 to 1.54)	1.59 (1.50 to 1.70)
Income	2.80 (2.65 to 2.96)	1.73 (1.63 to 1.84)	1.59 (1.49 to 1.70)	1.52 (1.43 to 1.62)	1.81 (1.69 to 1.93)
Country of birth	2.45 (2.30 to 2.61)	1.83 (1.71 to 1.95)	3.13 (2.92 to 3.35)	3.19 (2.98 to 3.41)	2.22 (2.05 to 2.39)
Intensive care admissions					
Education	2.94 (2.55 to 3.40)	2.72 (2.34 to 3.18)	1.62 (1.39 to 1.89)	1.58 (1.35 to 1.84)	1.55 (1.33 to 1.81)
Income	1.58 (1.38 to 1.80)	0.93 (0.80 to 1.09)	1.64 (1.41 to 1.90)	1.52 (1.31 to 1.77)	1.69 (1.44 to 1.99)
Country of birth	2.99 (2.57 to 3.47)	2.69 (2.31 to 3.14)	2.18 (1.85 to 2.57)	2.23 (1.89 to 2.63)	2.04 (1.70 to 2.46)
Deaths					
Education	12.09 (10.84 to 13.49)	6.64 (5.92 to 7.44)	1.09 (0.98 to 1.22)	1.09 (0.98 to 1.22)	1.13 (1.01 to 1.26)
Income	10.84 (9.76 to 12.05)	8.14 (7.27 to 9.12)	1.94 (1.72 to 2.19)	1.90 (1.68 to 2.14)	1.82 (1.61 to 2.07)
Country of birth	0.31 (0.27 to 0.35)	0.15 (0.13 to 0.17)	1.08 (0.93 to 1.25)	1.09 (0.94 to 1.26)	0.99 (0.85 to 1.16)

*Model 0: crude. Model 1: Model 0 adjusted for the other two socioeconomic variables. Model 2: Model 1 adjusted for sex and age. Model 3: Model 2 adjusted for sex, age and the Charlson Comorbidity Index. Model 4: Model 3 adjusted for family structure, type of house, civil status and region of residence.

In contrast, adjusting for comorbidities (Model 3) only lead to minor changes in the estimates. In the final model adding demographic factors (Model 4), country of birth remained the indicator displaying steepest gradient for hospitalisation admission (RII=2.22) and intensive care (RII=2.04), with weaker but significant gradients for education and income (RII=1.59–2.04). Deaths displayed a clear gradient by income (RII=1.82), but with only weak gradient by education (RII=1.13) and zero gradient for country of birth (RII=0.99).

## Discussion

This study of all confirmed first-wave SARS-CoV-2 cases in Sweden demonstrates marked and ubiquitous social gradients in subsequent severe COVID-19-related outcomes, with generally worse outcomes the lower the income, the shorter the education, or the more disadvantaged country of birth. Country of birth was the indicator displaying the strongest gradient in hospitalisation and intensive care, while only income displayed a strong independent gradient in COVID-19 deaths. Importantly, we found no support for pre-existing chronic diseases underpinning the inequalities. This finding suggests that inequalities in consequences of coronavirus infection are not merely rooted in already manifest inequalities in chronic disease as has been suggested previously^17–19^ and raises questions about inequitable management of confirmed COVID-19 cases in the Swedish healthcare system.

For education and particularly country of birth, the magnitude of social gradients decreased with increasing disease severity; steeper gradient for hospitalisation, slightly lower for intensive care admissions, and little to no gradient observed for deaths. These findings correspond to a few US studies.^23–25 32^ Although the present study did not explore the reasons behind this pattern, results specifically point to inequalities emerging in the process from confirmed disease to hospital admission, rather than during specialised care. Greater social inequities for health promotion and in primary rather than secondary healthcare match previous Swedish studies on general healthcare utilisation.^33 34^ These are possibly explained by disparities in health literacy, healthcare-seeking behaviour and healthcare access, which could result in disadvantaged populations reaching healthcare with more advanced COVID-19 disease. Such inequalities would be expected to be cumulative to any inequities in exposure and immunity^19^ as well as in testing. As inequalities at admission seemed to be decreasing with more severe outcomes, this could signify more equitable management of patients once admitted.

Income inequalities displayed a different overall pattern, with moderate but consistent gradients across all COVID-19 outcomes, which were not mitigated on admission to hospital. Despite the universal healthcare coverage and the low out-of-pocket patient fees in Swedish healthcare, previous Swedish reports have also found income-related inequalities in healthcare utilisation in Sweden.^33 34^ A relative lack of means could potentially contribute to individuals seeking healthcare at more advanced COVID-19 disease. As increased virus load also has been shown to predict COVID-19 survival,^35^ differential exposure to COVID-19 could also play a role. These could be linked to the risks in low-income occupations such as blue-collar service workers^36^ or to living in more crowded disadvantaged neighbourhoods.^29^ This finding should warrant research and policy actors to investigate financial barriers to optimal COVID-19 care in Sweden.

Our findings are particularly important to interpret in the light of robust disparities in mortality by country of birth in the total population of Sweden^17^ and Stockholm Region.^18^ These are studies with different target populations but comparable to our study when it comes to data sources, measures and context. Interestingly, while we were unable to find any lower survival among immigrants with COVID-19, which corresponds to results of a US study also covering a long time period,^25^ supplementary analyses restricting the analyses to deaths captured by the aforementioned Swedish studies indeed showed higher death rate among immigrants from LICs. This finding may reflect an evolvement of inequality patterns along the course of the pandemic, as has been suggested by Clouston *et al*.^15^ We therefore caution against generalising results from restricted periods of the pandemic, which worryingly represents a large share of the current evidence on social inequalities in COVID-19 outcomes.^12^ Further research into the temporal dynamics of social inequalities in COVID-19 outcomes is necessary, not the least considering the ongoing global rollout of COVID-19 vaccination across the world which again could be expected to change patterns.

The seemingly paradoxical finding of lower hospitalisation and intensive care admissions but higher death rate among the oldest old and those living in special accommodations (including nursing homes) likely reflects Sweden’s tragic and harshly criticised failure to protect the frail elderly in nursing homes from severe COVID-19 disease and death,^6 37^ with reports of lack of medical examination and avoidance of hospitalisation in favour of palliative care.^37^

### Methodological considerations

The study population comprised all confirmed COVID-19 cases in the first wave of the pandemic in Sweden. This is a considerable strength compared with studies from other countries based on non-random samples^23–25 32^ and restricted time periods.^23 24 32^ Nevertheless, it is important to emphasise that the population does not represent the total population of COVID-19 cases in Sweden. The limited testing capacity in the early stage of the pandemic and the reliance of voluntary self-testing outside the clinical setting may have contributed to systematically lower identification rate of cases among disadvantaged socioeconomic groups and healthier populations outside the attention of healthcare. The extent of this bias is however unknown.

We applied a summary measure of comorbidities, which, while suitable to study severe COVID-19 outcomes^21^ in Swedish register-based research,^28^ is based on diagnoses recorded in hospital setting and does not completely capture the influence of comorbidities on COVID-19 outcomes. For example, alternative summary measures, such as frailty indices, also predict severe COVID-19 outcomes.^38^ Summary measures such as the CCI also likely underestimate the differential prognostic value of specific diseases.^39^

Unaccounted for in the study is the competing risk for death, which could bias the inequality estimates of the hospitalisation and intensive care outcomes, particularly if disadvantaged populations die earlier and outside the hospital setting. Moreover, the metric used only captures relative, that is, ratio-based, inequalities, and does not display absolute, that is, difference-based, health inequalities.

## Conclusions and policy implications

Our findings point to widespread social gradients in severe COVID-19 outcomes among all Swedish first-wave SARS-CoV-2 cases, with no support for the inequalities being rooted in pre-existing disparities in chronic diseases. The findings instead suggest that inequitable outcomes of COVID-19 infection arise prior to hospital admission and may thereby be amenable to equitable management by the health system. Tailoring information dissemination to underserved populations and closer monitoring test-positive COVID-19 cases could potentially prove to be important measures to promote equitable COVID-19-related healthcare and outcomes during the sustained pandemic. Targeted efforts for the ongoing vaccination rollout may also be required to safeguard against inequitable vaccination adding to the inequities reported in this study. While both the specific patterns of COVID-19 inequalities and the possibilities for public health and healthcare responses are highly context-dependent, consideration of these recommendations is of global relevance.

**What is already known on this subject**

- Studies have shown a social patterning of COVID-19 outcomes but are mostly based on limited samples from restricted time periods.
- A prevalent hypothesis is that inequalities in severe consequences of SARS-CoV-2 infection, such as survival, are attributed to pre-existing disparities in chronic disease.

**What this study adds**

- Examining all confirmed COVID-19 cases of the first pandemic wave in Sweden, we demonstrate widespread social gradients in severe COVID-19 outcomes that are independent of comorbidities.
- Results suggest that social gradients in severe COVID-19 outcomes emerge already prior to hospital admission, which calls for equity-promoting strategies to monitor test-positive cases.

## Data Availability

Data may be obtained from a third party and are not publicly available.
