# Supplementary material for: Inequitable impact of infection: social gradients in severe COVID-19 outcomes among all confirmed SARS-CoV-2 cases during the first pandemic wave in Sweden
Source: J Epidemiol Community Health. 2021 Sep 14;76(3):261–7. doi: 10.1136/jech-2021-216778 (PMC8449839; doi:10.1136/jech-2021-216778)
Supplement: Supplementary data [file jech-2021-216778supp001.pdf]

Supplement 1

Table 1S. Crude and adjusted incident rate ratios (IRR) for COVID-19 mortality (until 7th May 2020)

	Deaths	
	Crude IRR	Adjusted IRR
Sex		
Men	1	1
Women	0.54 (0.52, 0.58)	0.44 (0.41, 0.48)
Age		
56-65	1	1
26-45	0.10 (0.07, 0.15)	0.12 (0.08, 0.17)
46-65	0.31 (0.24, 0.41)	0.37 (0.27, 0.49)
66-75	6.12 (5.18, 7.23)	3.68 (3.07, 4.42)
76-85	15.43 (13.23, 17.99)	7.84 (6.54, 9.40)
86-95	21.94 (18.83, 25.55)	11.57 (9.58, 13.97)
=>96	23.77 (19.58, 28.86)	12.44 (9.83, 15.74)
Co-morbidity index		
0	1	1
1-2	7.93 (7.02, 8.96)	1.78 (1.55, 2.05)
3-4	24.07 (21.30, 27.20)	2.46 (2.13 2.83)
=>5	17.19 (15.27, 19.36)	2.63 (2.28, 3.02)
Housing		
House	1	
Flat/Semidetached	1.88 (1.73, 2.04)	1.11 (1.02, 1.22)
Other	2.51 (1.94, 3.23)	1.16 (0.89, 1.51)
Special accommodation	9.98 (9.05, 11.01)	1.37 (1.22, 1.54)
Family structure		
Together w/out children	1	1
Together with children	0.10 (0.08, 0.12)	0.80 (0.65, 0.97)
Alone without children	2.00 (1.86, 2.16)	1.19 (1.05, 1.36)
Alone with children	0.27 (0.22, 0.34)	1.26 (0.97, 1.63)
Other without children	1.02 (0.89, 1.18)	1.21 (1.03, 1.43)
Other with children	0.41 (0.35, 0.49)	1.22 (0.99, 1.50)
Civil status		

Partner	1	1
Single	0.52 (0.47, 0.58)	0.91 (0.79, 1.06)
Widowed	6.11 (5.65, 6.61)	0.92 (0.80, 1.05)
Divorced	1.65 (1.50, 1.81)	0.89 (0.78, 1.02)
Region of residence		
Other	1	1
Västra Götaland	0.71 (0.63, 0.79)	0.52 (0.46, 0.59)
Stockholm	2.15 (2.01, 2.30)	1.07 (0.99, 1.15)
Education		
Tertiary	1	1
Secondary (10-12 years)	1.96 (1.79, 2.15)	1.13 (1.03, 1.25)
Primary (< 10 years)	5.01 (4.57, 5.49)	1.06 (0.96, 1.18)
Income		
Quintile 1 (richest)	1	1
Quintile 2	0.84 (0.72, 0.99)	1.08 (0.91, 1.28)
Quintile 3	1.59 (1.38, 1.83)	1.37 (1.17, 1.59)
Quintile 4	4.36 (3.86, 4.93)	1.50 (1.30, 1.73)
Quintile 5 (poorest)	4.42 (3.91, 4.99)	1.66 (1.43, 1.92)
Place of birth		
Sweden	1	1
High income countries	1.39 (1.26, 1.54)	0.99 (0.88, 1.10)
Middle income countries	0.47 (0.42, 0.53)	0.86 (0.73, 1.00)
Low income countries	0.61 (0.52, 0.72)	1.37 (1.10, 1.71)

Table 2S. Association between the socioeconomic variables and Charlson comorbidity index (CCI), using a cut-off of 5 and 3.*

	CCI (=>5)		CCI (=>3)	
Education	Crude IRR	Adjusted IRR	Crude IRR	Adjusted IRR
Tertiary	1	1	1	1
Secondary	1.07 (1.02, 1.13)	1.09 (1.04, 1.15)	1.09 (1.05, 1.13)	1.09 (1.05, 1.14)
Primary	1.14 (1.07, 1.20)	1.17 (1.10, 1.24)	1.17 (1.11, 1.22)	1.18 (1.13, 1.24)
Income				
Quintile 1 (richest)	1	1	1	1
Quintile 2	1.05 (0.97, 1.13)	1.05 (0.97, 1.13)	1.06 (0.99, 1.13)	1.07 (1.01, 1.14)
Quintile 3	1.22 (1.14, 1.31)	1.23 (1.14, 1.33)	1.24 (1.17, 1.32)	1.26 (1.19, 1.34)
Quintile 4	1.40 (1.31, 1.50)	1.41 (1.31, 1.51)	1.38 (1.31, 1.46)	1.40 (1.32, 1.48)
Quintile 5 (poorest)	1.31 (1.22, 1.40)	1.32 (1.22, 1.42)	1.36 (1.29, 1.44)	1.36 (1.29, 1.45)
Place of birth				
Sweden	1	1	1	1
High Income country	1.07 (1.00, 1.15)	1.01 (0.94, 1.09)	1.05 (0.99, 1.10)	1.00 (0.95, 1.06)
Middle income country	1.09 (1.03, 1.16)	1.05 (0.98, 1.12)	1.03 (0.98, 1.08)	1.01 (0.96, 1.07)
Low-income country	1.08 (0.98, 1.18)	1.03 (0.94, 1.14)	1.02 (0.94, 1.10)	0.99 (0.91, 1.08)

model 1 adjusted for age, sex; model 2: model 1 + civil status, house type, accommodation and place of residence. Two cut-off points, CCI=>5 and CCI=>3
